# APTX acts in DNA double-strand break repair in a manner distinct from XRCC4

**DOI:** 10.1093/jrr/rrad007

**Published:** 2023-03-20

**Authors:** Rikiya Imamura, Mizuki Saito, Mikio Shimada, Junya Kobayashi, Masamichi Ishiai, Yoshihisa Matsumoto

**Affiliations:** Laboratory for Zero-Carbon Energy, Institute of Innovative Research, Tokyo Institute of Technology, 2-12-1 Ookayama, Meguro-ku, Tokyo 152-8550, Japan; National Cancer Center Research Institute, 5-1-1 Tsukiji, Chuo-ku, Tokyo 104-0045, Japan; Laboratory for Zero-Carbon Energy, Institute of Innovative Research, Tokyo Institute of Technology, 2-12-1 Ookayama, Meguro-ku, Tokyo 152-8550, Japan; Laboratory for Zero-Carbon Energy, Institute of Innovative Research, Tokyo Institute of Technology, 2-12-1 Ookayama, Meguro-ku, Tokyo 152-8550, Japan; Department of Radiological Sciences, School of Health Science at Narita, International University of Health and Welfare, 4-3 Kozunomori, Narita, Chiba 286-8686, Japan; National Cancer Center Research Institute, 5-1-1 Tsukiji, Chuo-ku, Tokyo 104-0045, Japan; Laboratory for Zero-Carbon Energy, Institute of Innovative Research, Tokyo Institute of Technology, 2-12-1 Ookayama, Meguro-ku, Tokyo 152-8550, Japan

**Keywords:** APTX, DNA double-strand break repair, non-homologous end joining (NHEJ), abortive ligation, XRCC4, CRISPR/Cas9

## Abstract

Aprataxin (APTX), the product of the causative gene for hereditary neurogenerative syndromes Ataxia-oculomotor apraxia 1 and early onset ataxia with oculomotor apraxia and hypoalbuminemia, has an enzymatic activity of removing adenosine monophosphate from DNA 5′-end, which arises from abortive ligation by DNA ligases. It is also reported that APTX physically binds to XRCC1 and XRCC4, suggesting its involvement in DNA single-strand break repair (SSBR) and DNA double-strand break repair (DSBR) via non-homologous end joining pathway. Although the involvement of APTX in SSBR in association with XRCC1 has been established, the significance of APTX in DSBR and its interaction with XRCC4 have remained unclear. Here, we generated *APTX* knock-out (*APTX*^−/−^) cell from human osteosarcoma U2OS through CRISPR/Cas9-mediated genome editing system. *APTX*^−/−^ cells exhibited increased sensitivity toward ionizing radiation (IR) and Camptothecin in association with retarded DSBR, as shown by increased number of retained γH2AX foci. However, the number of retained 53BP1 foci in *APTX*^−/−^ cell was not discernibly different from wild-type cells, in stark contrast to XRCC4-depleted cells. The recruitment of GFP-tagged APTX (GFP-APTX) to the DNA damage sites was examined by laser micro-irradiation and live-cell imaging analysis using confocal microscope. The accumulation of GFP-APTX on the laser track was attenuated by siRNA-mediated depletion of XRCC1, but not XRCC4. Moreover, the deprivation of APTX and XRCC4 displayed additive inhibitory effects on DSBR after IR exposure and end joining of GFP reporter. These findings collectively suggest that APTX acts in DSBR in a manner distinct from XRCC4.

## INTRODUCTION

Genotoxic stress such as ionizing radiation (IR) and chemical compounds can induce a variety of DNA damages, including base damages, single-strand breaks (SSBs) and double-strand breaks (DSBs). The damages are repaired promptly by various DNA repair mechanisms depending on the types of DNA damage, such as base excision repair (BER), SSB repair (SSBR) and DSB repair (DSBR), through homologous recombination repair (HRR), non-homologous end joining (NHEJ) and microhomology-mediated end joining (MMEJ, also called alternative end-joining or theta-mediated end joining) pathway [1–3]. If unrepaired or repaired incorrectly, DNA damages cause cell death or carcinogenesis. Therefore, the mutations or defects in genes of DNA repair components result in genetic disorders with pleiotropic symptoms, including immunodeficiency, growth defect, neurodegeneration and/or increased cancer predisposition at organ level, as well as elevated sensitivity to the genotoxic agent and increased frequency of chromosome aberration at cellular level [3–6].

Aprataxin (APTX) is an enzyme that removes adenosine monophosphate (AMP) from DNA 5′-end, resolving adenylation of nicked DNA, single/double-strand DNA end and RNA/DNA junction during abortive DNA ligation by DNA ligases [7–11]. Human has three DNA ligases, *i.e*. DNA ligase IV involved in DSBR though NHEJ pathway, DNA ligase I (LIG1) and DNA ligase III (LIG3) involved in SSBR and DSBR though MMEJ pathway [2, 12, 13]. In addition, certain isoforms of APTX, with N-terminal extension of 14 amino acids, localize not only to the nucleus but also to the mitochondria and maintain its function [14, 15]. Furthermore, mutations in *APTX* gene cause recessively inherited neurodegenerative disorders, such as ataxia oculomotor apraxia 1 (AOA1) and early onset ataxia with oculomotor apraxia and hypoalbuminemia (EAOH) [16, 17]. The accumulation of abortive ligation might contribute to the pathogenesis of AOA1 and EAOH, because DNA ligation is the final process in almost all DNA repair pathway. AOA1 patient-derived cells exhibit moderate sensitivity to the genotoxic agents such as methyl methanesulphonate (MMS) and hydrogen peroxide (H_2_O_2_) but, at most, mild sensitivity to IR [18–21]. In a mouse model, *Aptx* knock-out mice do not display overt phenotype and neural cells from these mice have almost normal DNA repair function [7, 22]. APTX is composed of the N-terminal forkhead-associated (FHA) domain, the central histidine triad (HIT) domain and the C-terminal zinc finger domain, mediating interaction with other proteins, catalytic activity and DNA binding, respectively [8, 9, 23]. X-ray cross-complementing 1 (XRCC1) and X-ray cross-complementing 4 (XRCC4) are key scaffold proteins that play essential roles in SSBR and DSBR via NHEJ pathway, respectively [2, 3, 24]; the mutation of these proteins also results in inherited diseases with neurodegeneration, growth defect and microcephaly, respectively [3, 25]. Since APTX through FHA domain physically binds to threonine-phosphorylated XRCC1 and XRCC4 (Thr519 and Thr233, respectively) by Casein kinase II, APTX is thought to be involved in SSBR and DSBR via NHEJ pathway [18, 19, 23, 26, 27]. Early studies have demonstrated the function of APTX in association with XRCC1 in SSBR in cell [18, 28–30]. However, the significance of APTX and its interaction with XRCC4 in DSBR in cell are still poorly understood.

In this study, we generated *APTX*^−/−^ U2OS cells through CRISPR/Cas9-mediated genome editing and show that these cells are deficient for DSBR. XRCC4 interacts with APTX but is dispensable for the recruitment of APTX to DNA damage induced by the laser micro-irradiation in living cells. Moreover, analyses of γH2AX after IR exposure and end joining of GFP reporter indicated distinct roles of APTX and XRCC4 in DSBR.

## MATERIALS AND METHODS

### Cell culture and drug treatment

The human osteosarcoma cell line U2OS was obtained from the American Type Culture Collection and U2OS *APTX*^−/−^ cell line was established in this study (see below). Cells were maintained in Dulbecco’s modified Eagle’s medium (DMEM; Nacalai Tesque Inc) supplemented with 10% v/v fetal bovine serum (FBS; Hyclone in GE Healthcare), 100 units/ml penicillin and 100 μg/ml streptomycin (Nacalai Tesque Inc) at 37°C in humidified atmosphere containing 5% CO_2_ conditions. Cell lines were regularly tested for mycoplasma contamination using e-MycoTM mycoplasma detection polymerase chain reaction (PCR) kit (iNtRON Biotechnology, Inc, Cat. No.# 25235). Where indicated, hydrogen peroxide (H_2_O_2_; Nacalai Tesque Inc) and Camptothecin (CPT; Sigma) were added to the culture medium at the final concentration of 0.5 mM and 1 μM, respectively.

### γ-ray irradiation

Cells were irradiated with γ-ray using ^60^Co source (222 TBq as of February 2010) in Chiyoda Technol Cobalt 60 Irradiation Facility, Laboratory for Zero-Carbon Energy, Institute of Innovative Research, Tokyo Institute of Technology. The dose rate was measured using an ionizing chamber-type exposure dosimeter C-110 (Oyo Giken) and corrected for decay by calculation.

### Colony formation assay

Cell survival after irradiation was measured by the colony formation assay. U2OS wild-type (WT) and *APTX*^−/−^ cells were plated on 6-well plates. Numbers of cells plated to each well were increased for higher doses of radiation so as to obtain appropriate number of colonies. After incubation for 12–14 h at 37°C under 5% CO_2_ conditions, cells were exposed to grading doses of γ-ray (1, 2 and 4 Gy) or treated with grading concentrations of CPT (7.5, 15 and 30 nM) for 24 h. Cells were further incubated for 10–14 days to form colonies. After washing with phosphate-buffered saline (PBS), cells were fixed with 99.5% ethanol and stained with 0.02% w/v crystal violet in 50% v/v methanol. After washing the plates with water and drying them overnight, colonies consisting of >50 cells were counted manually. Plating efficiency was calculated as the number of colonies divided by the number of plated cells. Surviving fraction was calculated as the plating efficiency of irradiated cells divided by that of unirradiated cells. Experiments were repeated at least three times independently.

### Construction of plasmid DNA

pEGFP-C1 was purchased from Clontech. Full-length human APTX cDNA was obtained by PCR from the cDNA pool of U2OS cells and inserted into pEGFP-C1. DNA constructs were verified by DNA sequence analysis. All primers are shown in Supplementary Table 1.

### Plasmid vector and siRNA transfections

For the plasmid vector transfection, PEI-MAX (Polysciences) was used according to the manufacturers’ instruction. For siRNA transfections, Lipofectamine RNAiMAX (Invitrogen in Thermo Fisher Scientific) was used according to the manufacturer’s instruction. All siRNAs were used at the final concentration of 50 nM. Typically, cells were subjected to analysis 48–72 h after plasmid vector or siRNA transfections. The sequences of the siRNA oligonucleotides are shown in Supplementary Table 2.

### Genome editing by CRISPR/Cas9 system and establishment of *APTX*  ^−/−^ cell lines

For the establishment of *APTX*^−/−^ cells using CRISPR/Cas9 system, the sgRNA target sequences in APTX gene were inserted into the pSpCas9(BB)-2A-Puro (PX459) V2.0 plasmid vector (Addgene) and verified by DNA sequencing. U2OS cells were transfected with the vectors and incubated for 2 days before the addition of selective agent, *i.e.* 2.0 μg/ml of puromycin (Invivogen). Five days later, clonal cells were isolated by plating 0.5 cells per each well of 96-well plates. APTX expression of each clone was examined by western blotting. To verify the presence of mutations in both *APTX* alleles, genomic DNA was prepared from each clone and the region containing *APTX* target was amplified by PCR, inserted into the pEGFP-C1 vector and sequenced. The sequences of APTX sgRNA and sequencing primers are shown in Supplementary Table 1.

### Establishment of GFP-APTX stably expressing cell lines

To establish cell lines stably expressing GFP-APTX, the media was replaced with fresh growth media containing 800 μg/ml Geneticin G418 (Nacalai Tesque Inc) on the subsequent day of the transfection. Seven days after transfection, clonal cells were isolated by plating 0.5 cells per each well of 96-well plates. GFP-APTX expression in each clone was examined by western blotting and fluorescence microscopy.

### Laser micro-irradiation and live-cell imaging

Laser micro-irradiation with confocal microscopy was performed as described by Tsukada *et al*. [31] with some modifications. Briefly, U2OS cells stably expressing GFP-APTX were plated on the glass-bottom 35 mm dishes. On the day before observation, culture media were replaced with phenol red-free DMEM (Nacalai Tesque Inc) supplemented with 10% v/v FBS and then 50 μM Xanthotoxin (also called 8-MOP, Tokyo chemical industry) was added to sensitize DNA to laser light. Leica TCS SP8 LIGHTNING Confocal Microscope (Leica microsystems) with a 63×/1.40 oil immersion objective lens was used for the induction of localized DNA damages by laser micro-irradiation, live-cell observation and capturing pictures. For the quantification, 15 cells were examined in each experiment. Time-lapse imaging was started before the laser micro-irradiation. Localized DNA damage was induced by irradiation of 405 nm laser at 100% intensity for 0.5 s and 488 nm light was used for the observation of GFP-APTX. The green fluorescence intensity at the irradiated area was quantitatively measured by using Leica SP-8 LASX software (Leica microsystems). The relative green fluorescence intensity was acquired after subtraction of the background intensity in the cells and division by the intensity at the irradiated area.

### SDS-PAGE and western blotting

Cells were lysed in the radioimmunoprecipitation assay buffer (50 mM Tris HCl, pH 7.5, 150 mM NaCl, 1 mM ethylenediaminetetraacetic acid (EDTA), 0.5% v/v Triton X-100, 0.1% w/v sodium dodecyl sulfate (SDS), 0.1% w/v sodium deoxycholate) containing protease inhibitor cocktail (Nacalai Tesque Inc, Cat. No. 25955–11) and phosphatase inhibitor cocktail (Nacalai Tesque Inc, Cat. No. 07575–51), and the protein concentration was measured by the bicinchoninic acid assay kit (Takara Bio) using bovine serum albumin (BSA) as the standard. In all experiment, 20 μg of protein was loaded onto SDS polyacrylamide gel electrophoresis (SDS-PAGE) plates. The proteins were electrophoresed at 40 mA/gel plate for 1.5 h, and transferred onto a polyvinylidene fluoride (PVDF) membrane at 100 V for 1.5 h. Next, the PVDF membrane was blocked with either 1% w/v BSA/TBS-T (tris-buffered saline containing 0.05% v/v Tween 20) or 1% w/v skim milk/TBS-T for 1 h at room temperature on a shaker. Then, the membrane was reacted for 2 h at room temperature or overnight at 4°C with the following primary antibodies diluted in 1% w/v BSA/TBS-T or 1% w/v skim milk/TBS-T: APTX (mouse, 1:750, Santa Cruz Biotechnology, Cat. No. sc-374 108), XRCC1 (mouse, 1:1000, Invitrogen in Thermo Fisher Scientific, Cat. No. MA5–13412), XRCC4 (rabbit, 1:500) [32], Ku80 (mouse, 1:2000, abcam, Cat. No. ab3715), PARP1 (mouse, 1:750, Santa Cruz Biotechnology, Cat. No. sc-74 470), GFP (mouse, 1:5000, Nacalai Tesque Inc, Cat. No. GF200) and KAP1 (rabbit, 1:4000, abcam, Cat. No. ab10484). After washing three times with TBS-T, the membrane was reacted for 1 h at room temperature with the secondary antibody, *i.e*. horseradish peroxidase-conjugated antibodies against rabbit or mouse immunoglobulins (1:3000, Dako, Cat. No. P0399 or P0447, respectively) diluted in 1% w/v BSA/TBS-T or 1% w/v skim milk/TBS-T. After washing six times with TBS-T, the membrane was developed by enhanced chemiluminescence (LI-COR, Biosciences) and detected by C-digit (LI-COR, Biosciences). For the quantification of the protein expression level, the density of the band was measured using ImageJ software.

### Immunoprecipitation

For the preparation of the samples for immunoprecipitation (IP), U2OS cells, which were grown on 10 cm dish, were washed in PBS (Nacalai Tesque Inc) and lysed in the lysis buffer (50 mM Tris–HCl, pH 7.5, 150 mM NaCl, 0.2% v/v NP-40, 1 mM EDTA, 10% v/v glycerol) supplemented with cocktails of protease inhibitors and phosphatase inhibitors. After incubation for 30 min with mixing on the rotator at 4°C, lysates were cleared by the centrifugation at 20 000 × g for 20 min at 4°C. Next, the cleared lysates were incubated with 10 μl of GFP-Trap magnetic agarose beads (ChromoTek, GmbH) for 2 h with mixing on a rotator at 4°C. The beads were then washed five times with the lysis buffer and proteins bound to beads were eluted in 2× SDS sample buffer (125 mM Tris–HCl, pH 6.8, 4% w/v SDS, 20% v/v glycerol, 0.01% w/v bromophenol blue, 5% v/v 2-mercaptoethanol).

### Immunofluorescence

Cells were grown on glass coverslips and pre-extracted with CSK buffer (10 mM piperazine-N,N′-bis(2-ethanesulfonic acid) (PIPES) KOH, pH 6.7, NaCl 100 mM, 300 mM sucrose, 1 mM ethyl glycol-bis(β-aminoethyl ether)-N,N,N′,N′-tetraacetic acid (EGTA), 3 mM MgCl_2_) containing 0.2% v/v Triton X-100 for 2 min at room temperature. Subsequently, cells were fixed and permeabilized with 4% w/v paraformaldehyde in PBS containing 0.2% v/v Triton X-100 for 10 min at room temperature. After 1 h of blocking in PBS-T (PBS containing 0.1% v/v Tween 20) supplemented with 1% w/v BSA, the following primary antibody reactions were performed in PBS-T supplemented with 1% w/v BSA for 2 h at room temperature: γH2AX (mouse, 1:2000, Merck Millipore, Cat. No. JBW301), 53BP1 (rabbit, 1:3000, Bethyl, Cat. No. A300-272A), PAN ADP-ribose binding reagent (rabbit,1:2000, Merck, Cat. No. 9QQ12P). Cells were washed three times with PBS-T, and secondary antibody reactions with Alexa Fluor 555-conjugated mouse or 488-conjugated rabbit secondary antibody (1:2000, Invitrogen in Thermo Fisher Scientific, Cat. No. A28180 or Cat. No. A32731, respectively) were performed in PBS-T supplemented with 1% w/v BSA for 1 h at room temperature in the dark. After five times washing with PBS-T, coverslips were stained with 100 ng/ml dye 4′,6-diamidino-2-phenylindole dihydrochloride in PBS-T for 30 min at room temperature in the dark and mounted in mounting medium (Dako).

Images were taken using Axio Observer microscope (Zeiss) with 20× dry or 63× water objective and prepared in Zen software v3.1 (Zeiss, blue edition). For the quantification of γH2AX, 53BP1 and ADP-ribose, the number of foci or the fluorescence intensity in each cell was measured using Cellprofiler software [33]. At least 300 cells were analyzed randomly from the pool of three independent experiments.

### End joining assay

End joining assay was performed as described in Zhou *et al.* [34] with some modifications. We used EJ-U2OS cells, in which the end joining substrate pEJ [35] was integrated [34]. To induce DSB, the I-*Sce*I expression vector, pCBASce [36], was introduced to EJ-U2OS cells using PEI-MAX after siRNA transfection. The percentage of GFP-positive cells was quantified by flow cytometric analysis using Cell lab Quanta SC (Beckman Coulter) 2 days after the vector transfection.

### Statistical analysis

Statistical analysis was performed using either GraphPad Prism 9 (GraphPad Software Inc) or Microsoft Excel. Unpaired, two-tailed *t*-test was applied to analyze the statistical significance of difference between two experimental groups. Sample sizes are indicated in figure legends. All experiments were independently performed at least two times, with similar results. In all experiments, the difference between two experimental groups was considered statistically significant when *P* < 0.05.

## RESULTS

### Generation of *APTX*  ^−/−^ U2OS cells through genome editing by CRISPR-Cas9

**Fig. 1:**
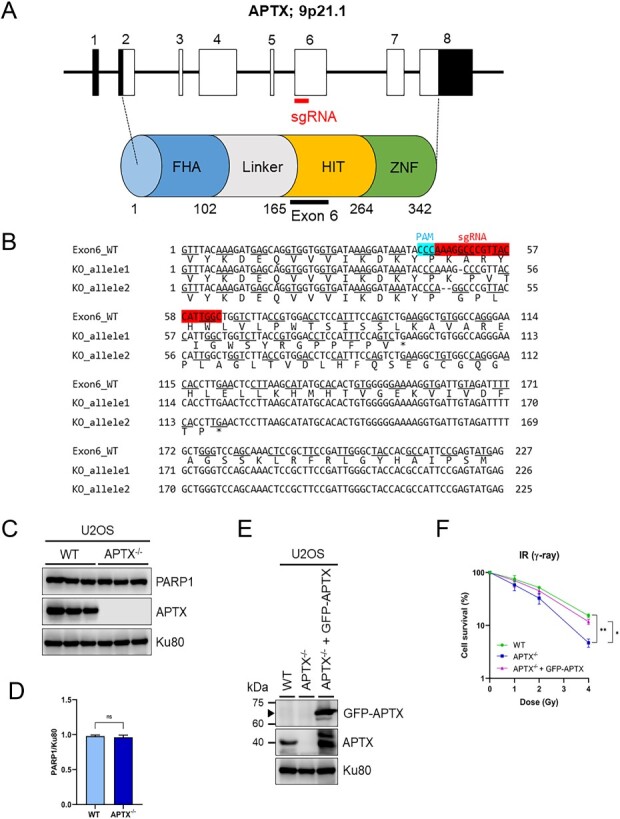
Establishment and characterization of *APTX*^−/−^ U2OS cell. (**A**) The human genomic *APTX* locus, sgRNA target for CRISPR/Cas9-mediated genome editing and the domain structure of the protein. The boxes represent exons. Open parts represent the open reading frame and filled parts represent 5′- or 3′-untranslated regions. The sgRNA target is located on exon 6, corresponding to HIT domain of APTX. (**B**) DNA sequence of two knocked out alleles of *APTX* exon 6 in *APTX*^−/−^ U2OS cells with reference to the original sequence shown at the top. PAM and sgRNA sequences are shown in light blue and red, respectively. Two alleles showed 1 and 2 bp deletion, respectively, as indicated by ‘-’, which will result in the frameshift. Each sequence includes the amino acid corresponding to its codon at the bottom. ‘^*^’ represents the stop codon. (**C**) Western blotting analyses of APTX and PARP1 proteins in WT and *APTX*^−/−^ U2OS. Each lane represents independent sample from biological replicate. Ku80 was shown as the loading control. (**D**) The quantification of PARP1 expression normalized by Ku80 expression in WT and *APTX*^−/−^ U2OS cell. Each columns represent the mean + SEM of three replicates. Statistical analysis was conducted by unpaired *t*-test assuming equal variance. ns = not significant, *P* > 0.05. (**E**) Western blotting analyses of APTX and GFP-APTX proteins in WT, *APTX*^−/−^ and *APTX*^−/−^ + GFP-APTX U2OS cells. The filled arrowhead represents predicted molecular mass of GFP-APTX, *i.e.* 68 kDa. Ku80 was shown as the loading control. (**F**) The IR sensitivity of WT, *APTX*^−/−^ and *APTX*^−/−^ + GFP-APTX cell measured by colony formation assay. Cell survival is expressed as a percentage relative to the untreated control, and the data represent the means ± SEMs of three replicates for each condition. Statistical analysis was performed by unpaired *t*-test assuming equal variance. ^*^0.01 < *P* ≦ 0.05; ^*^^*^0.005 < *P* ≦ 0.01.

We thought it difficult to examine APTX function using AOA1 patient-derived cells, because there are >20 mutations in different genetic backgrounds [37]. Therefore, we sought to establish *APTX* knock-out U2OS (*APTX*^−/−^) cells by CRISPR-Cas9 genome editing targeted the exon 6 of *APTX* gene locus, which correspond to the HIT domain of Apratxin (Fig. 1A) [38, 39]. *APTX*^−/−^ cells showed frameshifts of both alleles in genomic DNA sequencing and complete absence of APTX protein in western blotting (Fig. 1B and C). In earlier studies, some of AOA1 patient-derived cells and *APTX* knock-out U2OS cells exhibited decreased PARP1 protein expression level [15, 28]. However, *APTX*^−/−^ cells generated here did not display appreciable reduction in PARP1 protein expression level (Fig. 1C and D).

We also constructed a plasmid vector to express GFP-tagged APTX (GFP-APTX) and transfected it transiently into *APTX*^−/−^ cells. The expression of GFP-APTX was confirmed by western blotting (Fig. 1E). An additional band detected below GFP-APTX was considered to be a degradation product of GFP-APTX (Fig. 1E). We measured IR (γ-ray) sensitivity of WT, *APTX*^−/−^ and *APTX*^−/−^ + GFP-APTX cells by colony formation assay. *APTX*^−/−^ cells showed decreased cell survival, especially after 4 Gy irradiation, compared with WT cells (Fig. 1F). On the other hand, *APTX*^−/−^ + GFP-APTX cells restored the cell survival to a level almost indistinguishable from that of WT cells (Fig. 1F). Treatment with a Topoisomerase I inhibitor CPT, which induces SSB and DSB in a manner dependent on DNA replication [40], also decreased the survival of *APTX*^−/−^ cells (Fig. S1). Although previous studies reported that AOA1 patient-derived cells displayed a mild sensitivity to IR exposure [18, 19], our results indicated that APTX is important for the survival of cells treated with IR or CPT.

### *APTX*  ^−/−^ cell is deficient for SSBR and DSBR

**Fig. 2:**
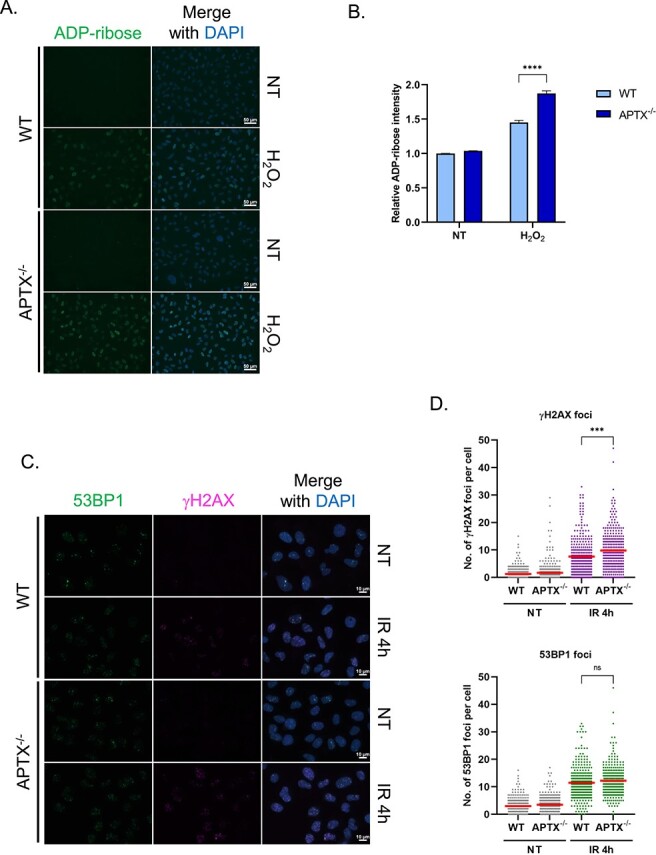
The measurement of SSBR and DSBR abilities in *APTX*^−/−^ cell. (**A**) Representative images of ADP-ribose staining in WT and *APTX*^−/−^ U2OS cells treated with or without 0.5 mM H_2_O_2_ for 10 min. (**B**) The quantification of ADP-ribose intensity normalized by the intensity of WT cell in non-treatment condition. Each column represents the mean + SEM of the pool from three independent sample preparation. Statistical analysis was conducted by unpaired *t*-test assuming equal variance. ^*^^*^^*^^*^0.0005 < *P* ≦ 0.001. (**C**) Representative images of γH2AX and 53BP1 foci in WT and *APTX*^−/−^ U2OS cells 4 h after 5 Gy γ-ray irradiation (IR). (**D**) The number of γH2AX and 53BP1 foci per cell. Dots represent the numbers of foci in respective cells and the red bars indicate the mean numbers of foci in respective experimental groups. The data from three independent experiments are pooled. Statistical analysis was performed by unpaired *t*-test assuming equal variance. ns = not significant, *P* > 0.05; ^*^^*^^*^: 0.001 < *P* ≦ 0.005.

We assessed SSBR abilities of *APTX*^−/−^ cells via ADP-ribosylation [41–43]. After treatment with hydrogen peroxide (H_2_O_2_), which induces base damage and SSB, for 10 min, *APTX*^−/−^ cells showed a significantly greater increase in ADP-ribose than WT cells (the mean of normalized ADP-ribose intensity was 1.9 and 1.5, respectively), indicating defective BER and/or SSBR in *APTX*^−/−^ cells and supporting the involvement of APTX therein (Fig. 2A and B).

We next evaluated the DSBR activity of *APTX*^−/−^ cells by immunostaining with γH2AX- and 53BP1-antibodies to probe DSB [44]. The number of γH2AX foci retained at 4 h after 5 Gy γ-ray irradiation in *APTX*^−/−^ cells was greater than that in WT cells (the mean of γH2AX foci was 9.8 and 7.6, respectively), suggesting delayed DSBR in *APTX*^−/−^ cells (Fig. 2C and D). Surprisingly, however, the number of 53BP1 foci under the above condition was not discernibly different between *APTX*^−/−^ cells and WT cells (the mean of 53BP1 foci was 12.2 and 11.5, respectively), suggesting that the DSBR via 53BP1 pathway is mostly normal in *APTX*^−/−^ cells (Fig. 2C and D). We also assessed the repair ability for CPT-induced DSB of *APTX*^−/−^ cells. As in the case of IR, the γH2AX intensity after CPT treatment for 1 h was higher in *APTX*^−/−^ cells than in WT cells (the mean of normalized γH2AX intensity was 2.7 and 2.1, respectively), but 53BP1 intensity was not significantly different between *APTX*^−/−^ cells and WT cells (the mean of normalized 53BP1 intensity was 1.4 and 1.3, respectively) (Fig. S2A–D). These results indicated that *APTX*^−/−^ cells lack general DSBR ability.

### XRCC4 interacts with APTX but is dispensable for the regulation of APTX dynamics in response to DNA damage

**Fig. 3:**
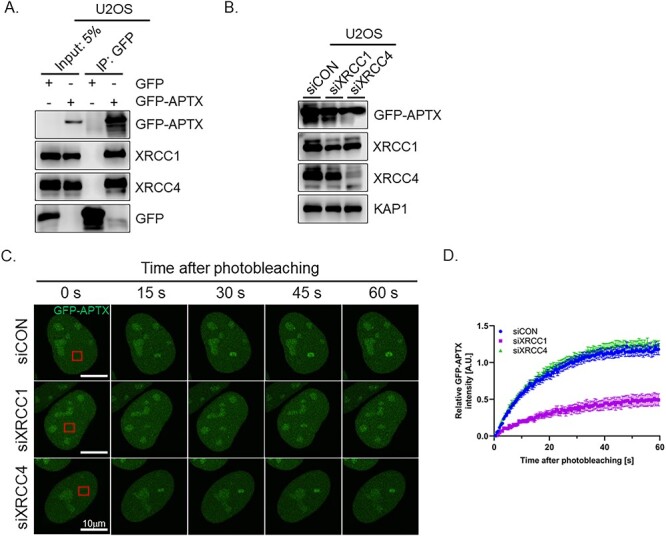
The interaction of APTX with XRCC1 and XRCC4 and their roles in the recruitment of APTX to DNA damage sites. (**A**) The interaction of GFP-APTX with XRCC1 and XRCC4 examined by IP. GFP-Trap magnetic agarose beads were used for IP and GFP, GFP-APTX, XRCC1 and XRCC4 proteins in the precipitate were examined by western blotting; 5% of whole cell lysate that was used in IP experiment is represented as input 5%. (**B**) The efficacy of knocking down XRCC1 and XRCC4 by siRNA. GFP-APTX stably expressing U2OS cells following the transfection of the indicated siRNAs were subjected to western blotting analysis of XRCC1, XRCC4 and GFP-APTX. KAP1 was shown as the loading control. The expression level of XRCC1 and XRCC4 normalized by KAP1 expression after siRNA treatment was indicated of 53 and 18% against siCON, respectively. (**C**) Representative live-cell images of the GFP-APTX stably expressing U2OS cells under the depletion of XRCC1 or XRCC4 after photobleaching. Micro-laser was irradiated on the area inside the red square. (**D**) Relative green fluorescent intensity of C. Fifteen cells were scored at each point. The data represent the mean ± SEM.

XRCC1 and XRCC4 bind to a variety of proteins and regulate the stabilization, enzymatic activity and dynamics of binding proteins [24, 30, 45–50]. We performed the IP in U2OS expressing GFP or GFP-APTX as bait. XRCC1 and XRCC4 were co-immunoprecipitated with GFP-APTX but not with GFP, indicating that APTX interacts with XRCC1 and XRCC4 in cell (Fig. 3A) as shown in earlier studies by others.

To analyze APTX dynamics in cell, GFP-APTX stably expressing U2OS cells were established and then depleted of endogenous XRCC1 or XRCC4 by small-interference RNA (siRNA) (Fig. 3B). We observed the recruitment of GFP-APTX to DNA damage site in live cell imaging using confocal microscopy with laser-irradiation system. GFP-APTX accumulated on the DNA damage sites and reached the plateau around 60 s after the induction in siControl (siCON) cell (Fig. 3C and D). XRCC1-depleted cells (siXRCC1) showed the attenuation of GFP-APTX recruitment to DNA damage sites compared with control cells (Fig. 3C and D) in agreement with previous study [30]. The residual recruitment of APTX might be due to remaining XRCC1 or other XRCC1-independent mechanism(s). On the other hand, there was not a discernible difference between the XRCC4-depleted (siXRCC4) and control cells. These results indicated that XRCC1, but not XRCC4, is necessary for the recruitment of APTX to DNA damage sites.

### Depletion of APTX and XRCC4 has an additive effect on DSBR

**Fig. 4:**
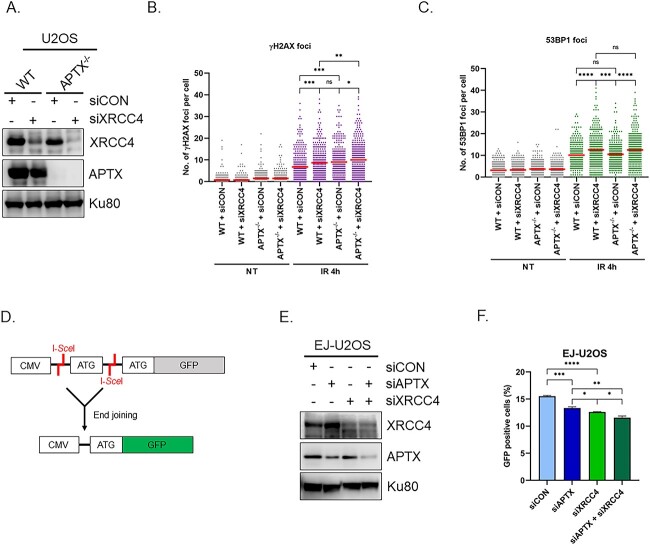
The epistatic relationship between APTX and XRCC4 in DSBR. (**A**) Western blotting analyses of XRCC4 and APTX in WT and *APTX*^−/−^ U2OS cells following transfection of the siXRCC4. Ku80 was shown as the loading control. (**B** and **C**) The quantification of γH2AX and 53BP1 foci in WT or *APTX*^−/−^ U2OS cells with siCON or siXRCC4 transfection 4 h after 5 Gy γ-ray irradiation (IR). Dots represent the numbers of foci in respective cells and the red bars indicate the mean numbers of foci in respective experimental groups. The data from three independent experiments are pooled. Statistical analysis was performed by unpaired *t*-test assuming equal variance. ns = not significant, *P* > 0.05; ^*^0.01 < *P* ≦ 0.05; ^*^^*^0.005 < *P* ≦ 0.01; ^*^^*^^*^0.001 < *P* ≦ 0.005; ^*^^*^^*^^*^0.0005 < *P* ≦ 0.001. (**D**) Schematic diagram of the end joining substrate. Translation of GFP is prevented by an insert between the CMV promoter and the start codon (ATG) of GFP, which is flanked by two inverted repeat I-*Sce*I recognition sequences including another start codon. Repair of the I-*Sce*I-induced DSB by end joining restores GFP translation. (**E**) Western blotting analyses of APTX and XRCC4 in EJ-U2OS cells following the transfection of siAPTX, siXRCC4 or both. Ku80 was shown as the loading control. (**F**) EJ-U2OS cells were transfected with I-*Sce*I expression vector and analyzed by flow cytometer. Each columns represent the mean + SEM from three independent sample preparation. Statistical analysis was performed by unpaired *t*-test assuming equal variance. ^*^0.01 < *P* ≦ 0.05; ^*^^*^0.005 < *P* ≦ 0.01; ^*^^*^^*^0.001 < *P* ≦ 0.005; ^*^^*^^*^^*^0.0005 < *P* ≦ 0.001.

Since the knock-down of XRCC4 did not affect the recruitment of APTX to DNA damage sites, we hypothesized that APTX has a role distinct from XRCC4 in DSBR. To examine the functional relationship between APTX and XRCC4 in DSBR, *APTX*^−/−^ and WT U2OS cells were depleted of XRCC4 by siRNA (Fig. 4A) and then tested for DSBR ability by γH2AX and 53BP1 immunostaining. As for γH2AX, *APTX*^−/−^ + siCON and WT + siXRCC4 conditions showed higher number of foci than the WT + siCON condition 4 h after irradiation (the mean of γH2AX foci was 8.7, 9.0 and 6.8, respectively) (Fig. 4B and C). Interestingly, *APTX*^−/−^ + siXRCC4 exhibited an additive increase in the number of γH2AX foci compared with *APTX*^−/−^ + siCON and WT + siXRCC4 (the mean of γH2AX foci was 10.7 in *APTX*^−/−^ + siXRCC4 cells). As for 53BP1, although WT + siXRCC4 and *APTX*^−/−^ + siXRCC4 showed remaining foci than WT + siCON and *APTX*^−/−^ + siCON (the mean of 53BP1 foci was 12.6, 12.5, 10.1 and 10.5, respectively), there was not a discernible difference between WT + siXRCC4 and *APTX*^−/−^ + siXRCC4.

To investigate the ability of end joining that is the final step in DSBR except for HRR, we used the end joining assay via GFP-reporter (Fig. 4D) [34, 35]. EJ-U2OS cells, in which GFP-reporter with substrates including I-*Sce*I recognition sequences had been integrated, were depleted of APTX, XRCC4 or both by siRNA (Fig. 4D and E). After the transfection of I-*Sce*I expressing vector, pCBASce, GFP-positive cells were counted by flow cytometric analysis. The fraction of GFP-positive cells was decreased by siAPTX and siXRCC4 in comparison to control siRNA (siCON), indicating APTX and XRCC4 are involved in end joining in DSBR (Fig. 4F). Moreover, the combination of siAPTX and siXRCC4 result in additive decrease in the fraction of GFP-positive cells in comparison to siAPTX and siXRCC4 (Fig. 4F).

These results support the above hypothesis that APTX and XRCC4 work in parallel in DSBR rather than the alternative possibility that they work in the same pathway.

## DISCUSSION

Wealth of *in vitro* biochemical studies have established the enzymatic activity of APTX removing AMP from 5′-end of DNA, which can arise from abortive DNA ligation. APTX has been shown to interact with XRCC1 and XRCC4, which are involved in SSBR and DSBR, respectively. There is accumulated evidence demonstrating an essential role of APTX in SSBR and the importance of its interaction with XRCC1 *in cellulo*. On the other hand, the role of APTX in DSBR and the importance of its interaction with XRCC4 *in cellulo* remain unclear.

Constitutive interaction between APTX and XRCC4 was initially identified by yeast two-hybrid screen and co-immunoprecipitation [19]. The FHA domain of APTX was shown to bind to XRCC4 even tighter, *i.e*. ~ 8-fold, than to XRCC1 in isothermal titration calorimetry experiment [23]. In the agreement with these studies, here we also showed co-immunoprecipitation of XRCC4 and GFP-APTX in U2OS (Fig. 3A). As it has been reported that the interaction of XRCC4 with XLF and PNKP promotes their accumulation of these NHEJ factors at DNA damage sites by laser-irradiation *in cellulo* [31, 49], it could have been inferred that APTX dynamics to DNA damage sites might also be regulated by XRCC4. In the present study, however, the accumulation of APTX to DNA damage sites was not affected by the depletion of XRCC4 by siRNA (Fig. 3C and D). One may have a concern that if the laser irradiation condition in this study does not induce DSBs effectively, the recruitment of APTX at DSB sites cannot be examined by this assay. However, it was shown that 405 nm laser irradiation in combination with BrdU or Xanthotoxin (8-MOP) produces DSB effectively, to which DSBR proteins such as Ku70 and Ku80 are recruited [51, 52]. Thus, we consider that the laser irradiation condition in this study must have induced DSBs to cells.

The present results also suggested that the role of APTX in DSBR might be different from that of XRCC4. First, the foci count and intensity of 53BP1, which are associated with DSBs, in *APTX*^−/−^ cells after IR exposure and CPT treatment were not discernibly different from WT control (Fig. 2C and D, Supplementary Fig. S2C and D), whereas siXRCC4 increased 53BP1 foci (Fig. 4C). Nevertheless, the foci count and intensity of γH2AX, which reflect DSBs, were increased in *APTX*^−/−^ cells, indicating that *APTX*^−/−^ cell is defective in DSBR (Fig. 2C and D, Supplementary Fig. S2A and B). Moreover, depletion of APTX and XRCC4 resulted in the additive effect on the persistence of γH2AX foci and end joining of GFP reporter (Fig. 4B and F). These results in the aggregate indicate that APTX acts in DSBR which is distinct from XRCC4-mediated NHEJ. The significance of the interaction between APTX and XRCC4, if any, remains to be clarified.

Then, how does APTX act in DSBR? LIG3 and LIG1 are implicated in DSBR through MMEJ as well as SSBR [13]. Considering the interaction of APTX with XRCC1, APTX might be involved in MMEJ via XRCC1 and LIG3. LIG1 is shown to have a unique feature of leaving the adenylated DNA 5′-end when DNA 3′-end includes a mismatch or oxidative base *in vitro* probably to prevent mutation, whose abortive ligation is safeguarded by APTX [10, 53]. In addition, it is also possible that MMEJ is retarded by APTX-deficiency.

Earlier studies by others have shown a lack of IR sensitivity in AOA1 patient-derived cells [18, 19]. However, *APTX*^−/−^ U2OS cells generated in this study showed increased sensitivity to IR exposure compared with parental WT U2OS cells (Fig. 1F). These disagreements might reflect different contribution of APTX to DNA repair depending on cell types. In fact, differential sensitivity to MMS or H_2_O_2_ treatment between lymphoblasts and fibroblasts from AOA1 patients has been reported [18, 19]. In addition, a series of mutants mimicking mutations in AOA1 patients exhibited different functionality in sensing nicked DNA and protein stability in *in vitro* assay [54]. Considering this, it is also possible that the IR sensitivity is also influenced by mutation sites of APTX.

The main symptoms of AOA1/EAOH include cerebellar ataxia, oculomotor ataxia and axonal/sensory neuropathy [3, 16, 17], which are similar to those of patients with XRCC1 mutation [3]. On the other hand, growth defect and microcephaly, which are manifested in patients with XRCC4 mutation, are not observed in AOA1/EAOH [25]. The requirement of XRCC1, but not XRCC4, for the recruitment of APTX to DNA damage sites (Fig. 3C and D) echoes these pathological conditions. Altogether, our results presented a novel role of APTX in DSB repair, which will bring us a deeper understanding for the mechanism of AOA1/EAOH pathogenesis.

## CONCLUSION

Here, we showed that the loss of APTX cells leads to defective DSBR and increased sensitivity to IR and CPT. We also demonstrated that XRCC4 is not required for the recruitment of APTX to DNA damage site. Finally, we showed that the deficiency of APTX and XRCC4 have additive effects on DSBR. These results in the aggregate pointed to the involvement of APTX in DSBR in a manner distinct from XRCC4.

## Supplementary Material

230102_APTX_Supplementary_figure_legends_rrad007

Supplementary_tables_230103_rrad007

Fig_S1_rrad007

Fig_S2A_B_rrad007

Fig_S2C_D_rrad007

## CONFERENCE PRESENTATION

Several parts of this study have been presented at the 64th and 65th Annual Meetings of Japanese Radiation Research Society and the 5th Asian Congress of Radiation Research.
