# Supplementary material for: APTX acts in DNA double-strand break repair in a manner distinct from XRCC4
Source: J Radiat Res. 2023 Mar 20;64(3):485–95. doi: 10.1093/jrr/rrad007 (PMC10214999; doi:10.1093/jrr/rrad007)
Supplement: 230102_APTX_Supplementary_figure_legends_rrad007 [file 230102_aptx_supplementary_figure_legends_rrad007.docx]

**Supplementary figure legends**

**Figure S1. the CPT sensitivity of APTX^-/-^ U2OS cell**

The CPT sensitivity of WT, APTX^-/-^ and APTX^-/-^ + GFP-APTX cell measured by colony formation assay. Cell survival is expressed as a percentage relative to the untreated control, and the data represent the means ± SEMs of three replicates for each condition. Statistical analysis was performed by unpaired t-test assuming equal variance. *: 0.01 < p ≦ 0.05; **: 0.005 < p ≦ 0.01.

**Figure S2. the measurement of CPT-induced DSBR ability in APTX^-/-^ U2OS cell**

A, representative images of γH2AX staining in WT and APTX^-/-^ U2OS cells treated with DMSO or 1 μM CPT for 1 h.

B, the quantification of γH2AX intensity normalized by the intensity of WT cell in non-treatment condition. Each column represents the mean + SEM of the pool from three independent sample preparation. Statistical analysis was conducted by unpaired t-test assuming equal variance. ****: 0.0005 < p ≦ 0.001.

C, representative images of 53BP1 staining in WT and APTX^-/-^ U2OS cells treated with DMSO or 1 μM CPT for 1 h.

D, the quantification of 53BP1 intensity normalized by the intensity of WT cell in non-treatment condition. Each column represents the mean + SEM of the pool from three independent sample preparation. Statistical analysis was conducted by unpaired t-test assuming equal variance. ns: not significant, p > 0.05.
