# Supplementary material for: APTX acts in DNA double-strand break repair in a manner distinct from XRCC4
Source: J Radiat Res. 2023 Mar 20;64(3):485–95. doi: 10.1093/jrr/rrad007 (PMC10214999; doi:10.1093/jrr/rrad007)
Supplement: Supplementary_tables_230103_rrad007 [file supplementary_tables_230103_rrad007.docx]

Supplementary table 1 Sequences of DNA oligonucleotides.

“F” and “R” indicate forward and reverse sequences, respectively.

| Name |  | DNA sequences (5’-3’) |
| --- | --- | --- |
| GFP-APTX | F | ACAAGTCCGGACTCAGATCTATGATGCGGGTGTGCTGGTTGG |
|  | R | TATCTAGATCCGGTGGATCCTCACTGTGTCCAGTGCTTCCTG |
| APTX sgRNA | F | CACCGCCAATGGTAACGGGCCTTT |
|  | R | AAACAAAGGCCCGTTACCATTGGC |
| APTX sequencing primer | F | ATCTCGAGTGTACGTGTCACACAGTCTG |
|  | R | GTGGATCCTCTGAGGATAATGAAGCTGC |

Supplementary table 2 Sequences of siRNAs.

"d" indicates deoxyribonucleotide.

siControl targets at mouse Lig4 sequence which not including a homology in human.

| Name | | Oligonucleotide (5’-3’) |
| --- | --- | --- |
| siControl (siCON) | sense | GUGUUUUAAGACAAAAACAdTdG |
|  | anti-sense | UGUUUUUGUCUUAAAACACdTdC |
| siXRCC1  3’-UTR | sense | GAAGUAUGUGCUAUACACAdTdT |
|  | anti-sense | UGUGUAUAGCACAUACUUCdTdT |
| siXRCC4  3’-UTR | sense | CUAUGUUUUCUAUUCAUUUdCdT |
|  | anti-sense | AAAUGAAUAGAAAACAUAGdTdC |
| siAPTX  3’-UTR | sense | CUACUCAUUUCCUAAAUUAdTdT |
|  | anti-sense | UAAUUUAGGAAAUGAGUAGdTdT |
