# Supplementary figures and images for: APTX acts in DNA double-strand break repair in a manner distinct from XRCC4
Source: J Radiat Res. 2023 Mar 20;64(3):485–95. doi: 10.1093/jrr/rrad007 (PMC10214999; doi:10.1093/jrr/rrad007)

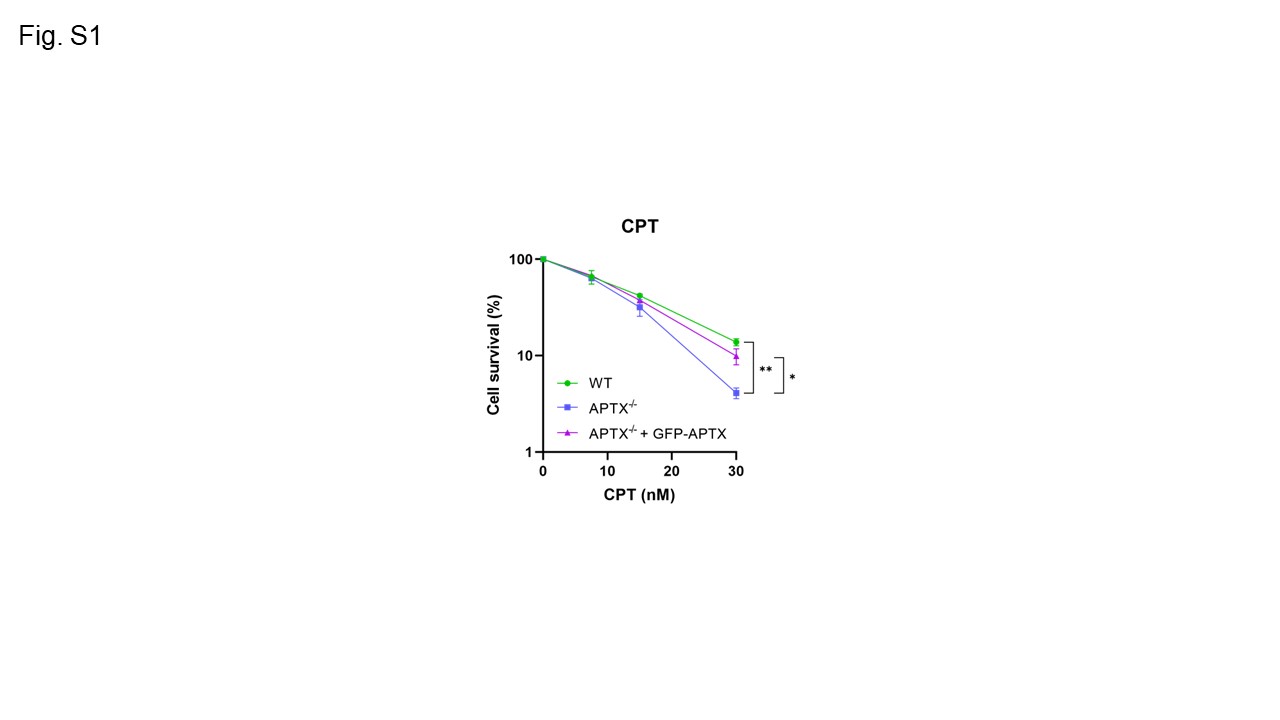

Supplement: Fig_S1_rrad007 [file fig_s1_rrad007.jpeg]

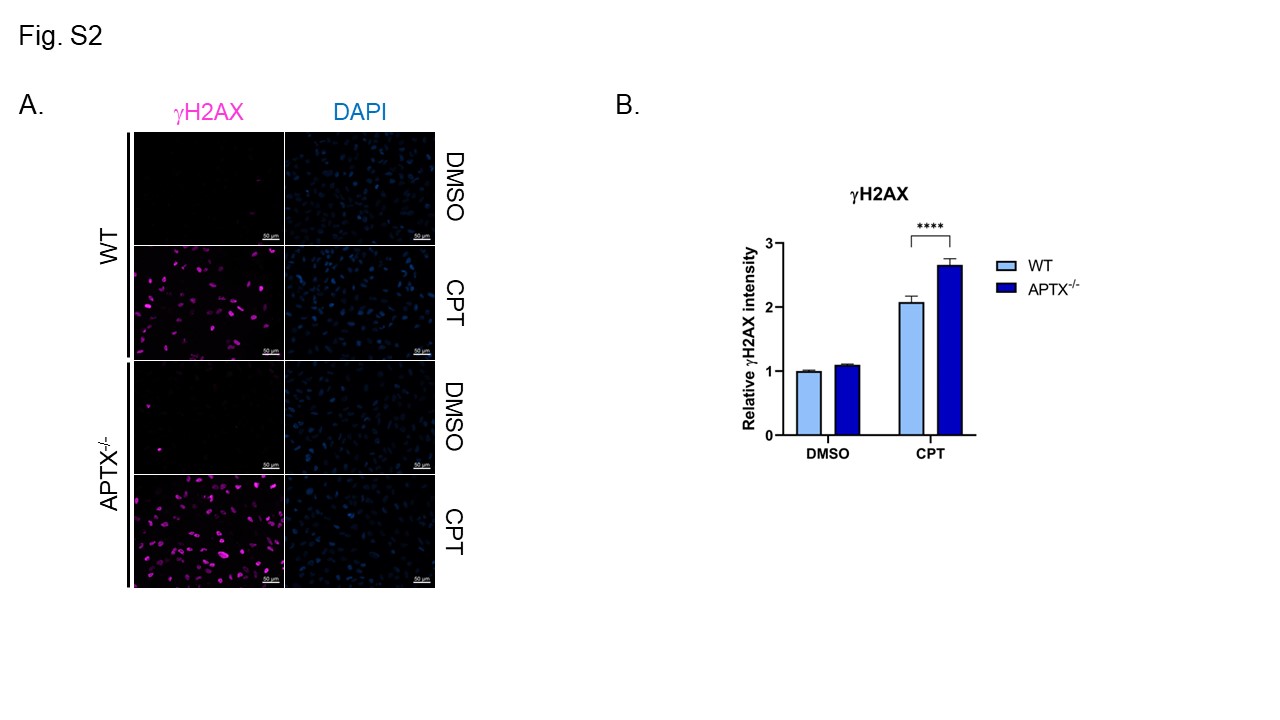

Supplement: Fig_S2A_B_rrad007 [file fig_s2a_b_rrad007.jpeg]

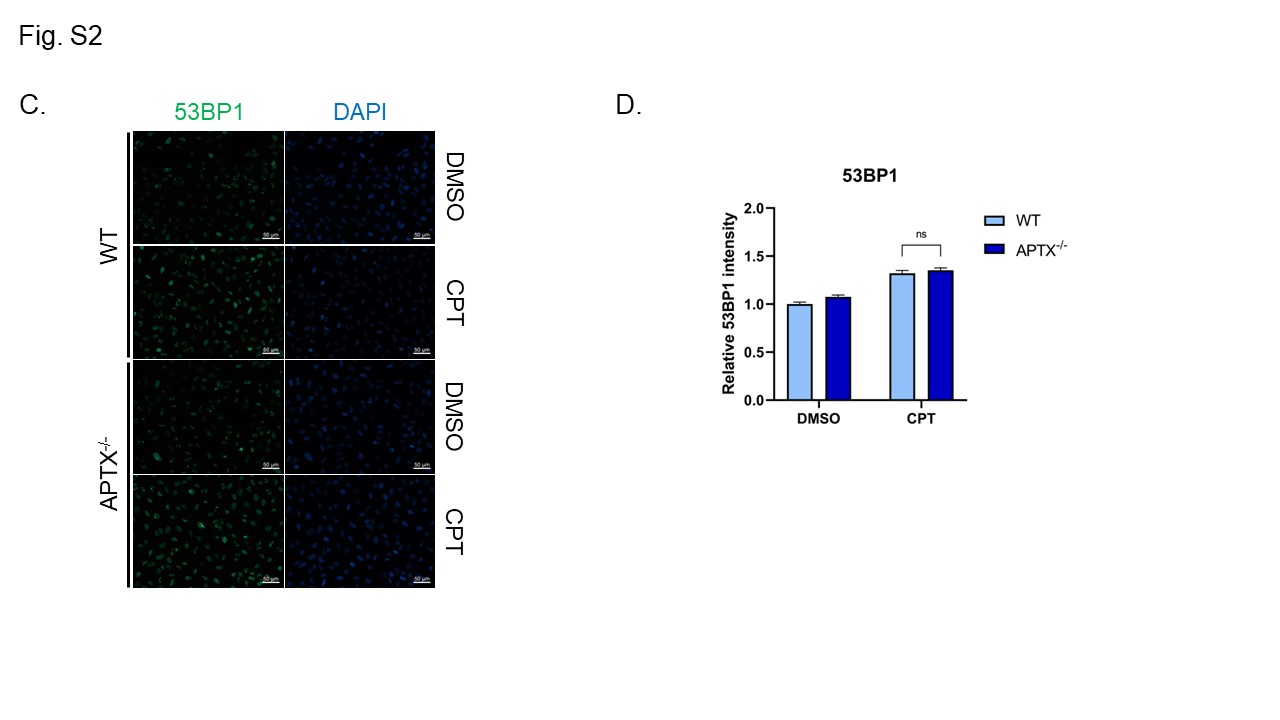

Supplement: Fig_S2C_D_rrad007 [file fig_s2c_d_rrad007.jpeg]
